# Characterization of anthropometric assessment studies of Brazilian children attending daycare centers

**DOI:** 10.1016/j.rppede.2016.01.002

**Published:** 2016

**Authors:** Dixis Figueroa Pedraza, Tarciana Nobre de Menezes

**Affiliations:** Universidade Estadual da Paraíba (UEPB), Campina Grande, PB, Brazil

**Keywords:** Anthropometrics, Children, Daycare centers, Systematic review

## Abstract

**Objective:**

To obtain an overview of available information on the anthropometric assessment of Brazilian children attending daycare centers.

**Data source:**

A literature search was carried out in the PubMed, LILACS and SciELO databases of studies published from 1990 to 2013 in Portuguese and English languages. The following search strategy was used: (*nutritional status* OR *anthropometrics* OR *malnutrition* OR *overweight*) AND daycare centers, as well as the equivalent terms in Portuguese. In the case of MEDLINE search, the descriptor *Brazil* was also used.

**Data synthesis:**

It was verified that the 33 studies included in the review were comparable from a methodological point of view. The studies, in general, were characterized by their restrictive nature, geographical concentration and dispersion of results in relation to time. Considering the studies published from 2010 onwards, low prevalence of acute malnutrition and significant rates of stunting and overweight were observed.

**Conclusions:**

Despite the limitations, considering the most recent studies that used the WHO growth curves (2006), it is suggested that the anthropometric profile of Brazilian children attending daycare centers is characterized by a nutritional transition process, with significant prevalence of overweight and short stature. We emphasize the need to develop a multicenter survey that will more accurately define the current anthropometric nutritional status of Brazilian children attending daycare centers.

## Resumo

**Objetivo:**

Traçar um panorama da informação disponível sobre a avaliação antropométrica de crianças brasileiras assistidas em creches.

**Fontes de dados:**

Pesquisa bibliográfica nas bases de dados PubMed, LILACS e SciELO de estudos publicados de 1990 a 2013 nos idiomas português e inglês. Foi utilizada a seguinte estratégia de busca: (*estado nutricional* OR *antropometria* OR *desnutrição* OR *sobrepeso*) AND *creches*. No caso da busca no MEDLINE o descritor *Brazil* também foi usado.

**Síntese dos dados:**

Verificou-se que os 33 estudos incluídos apresentaram comparabilidade do ponto de vista metodológico. Os estudos, no seu conjunto, caracterizaram-se por sua natureza restritiva, concentração geográfica e dispersão dos resultados em relação ao tempo. Considerando os estudos publicados a partir de 2010, observam-se baixas prevalências de desnutrição aguda e taxas expressivas de déficit de estatura e de sobrepeso.

**Conclusões:**

Apesar das limitações, considerando os estudos mais recentes que utilizaram as curvas de crescimento OMS (2006), sugere-se que o perfil antropométrico de crianças brasileiras assistidas em creches caracteriza-se por um processo de transição nutricional com prevalências expressivas de sobrepeso e de baixa estatura. Ressalta-se a necessidade do desenvolvimento de um inquérito multicêntrico para delimitar de forma mais precisa o estado nutricional antropométrico atual das crianças brasileiras que frequentam creches.

## Introduction

Nutritional status has a significant influence on morbidity and mortality and the process of child growth and development. Thus, the assessment of the nutritional status of the Pediatric population is essential to identify the appropriate interventions to improve health and life conditions.^1^ Anthropometrics is the most universally used method to assess children's nutritional status. It stands out because it is an easily applied, low cost and non-invasive method, as well as objective and sensitive.^2^^,^ 3

Child malnutrition remains one of the most important public health problems in the world today, due to its magnitude and devastating consequences for children's growth, development and survival.^4^ National surveys on health and nutrition show an ongoing decrease in cases of malnutrition in Brazil. This improvement is attributed to the favorable evolution of socioeconomic and health care conditions, so that the adequate nutrition of the poorest segments of society remain a major challenge for public policies in Brazil.^5^^,^ 6

The benefit offered by daycare centers is considered an important strategy of developing countries to improve growth and development of children belonging to lower social strata. The demand for these services is high, due to the growing participation of women in the labor market, especially in large and medium cities in Brazil, with an increase in the number of daycare centers and assisted children.^7^ Therefore, daycare centers have been gradually changing by turning into public policy proposal in the education, nutrition and health care areas. The benefit constitutes the main public policy instrument aimed at promoting food and nutrition security of the urban population of infants and preschool children from low income families.^8^ However, the increase in infectious diseases and the non-compliance with standards that regulate the care of children in daycare centers are factors that have been reported, with possible negative effects on the attainment of the program objectives.^8^^,^ 9 Methodologically, the tendency for children attending daycare centers to improve their nutritional status and/or to acquire more infectious diseases can be determined through anthropometric indicators, viable and safe predictors of health status, functional impairment and mortality.^10^

The identification of the anthropometric profile of children attending daycare centers is, therefore, a key step in the design and/or redesign of actions in day care centers that aim to promote adequate nutritional status and overall health of the assisted children. Given the above, this study aims to provide an overview of available information on the anthropometric assessment of Brazilian children attending daycare centers.

## Method

A search was carried out in PubMed (National Library of Medicine, Bethesda, MD), LILACS (Latin American and Caribbean Health Sciences) and SciELO (Scientific Electronic Library Online) databases. The search was carried out on January 3, 2014, using the following strategy: (*nutritional status* OR *anthropometrics* OR *malnutrition* OR *overweight*) AND daycare centers, as well as the equivalent terms in Portuguese. In the PubMed search, the descriptor *Brazil* was also used. We chose to search for studies published since 1990, using the Portuguese and English languages.

To calculate the total number of the identified studies, their duplication or triplication was verified among the databases and each item was counted only once. The decision on the inclusion of articles included two steps: (a) screening by reading the titles and abstracts, (b) reading of the full text. In the screening phase, intervention studies, review studies, books or thesis, studies performed out of Brazil and studies of children not attending daycare centers were eliminated. At the full-text reading phase, observational studies with representative samples that analyzed anthropometric indices (height *z*-score for age, weight *z*-score for height, weight *z*-score for age, body mass index) of Brazilian children attending daycare centers were included. Studies with unrepresentative and/or non-randomly selected samples, based on the analysis of secondary data, and showing no results of the prevalence of malnutrition and/or overweight, were excluded.

The included studies were organized according to the study location into four groups: (a) national studies and South, Southeast, Midwest and North studies; (b) studies in the Northeast region; (c) studies in other cities rather than the capital of the state of São Paulo; (d) studies in the capital city of São Paulo. The characterization of the studies was performed according to the author and year of publication, location, anthropometric assessment method, reference standard and outcomes (prevalence of anthropometric deviations).

## Results

**Figure 1:**
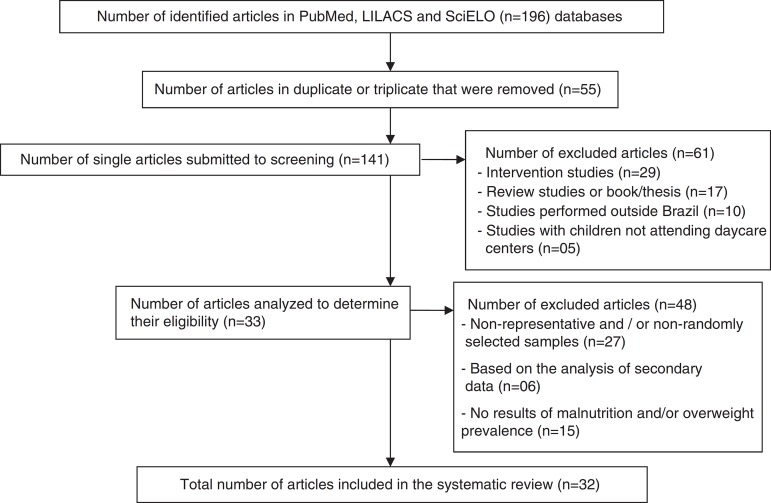
Flowchart of the phases of identification, screening and selection of articles on anthropometric evaluation of Brazilian children attending daycare centers.

A total of 141 records were identified in searched databases, which were submitted to screening. After analyzing the titles and abstracts, 61 articles that did not meet the selection criteria were excluded. Subsequently, after the full reading of the 80 eligible articles, 48 were excluded because they met some of the exclusion criteria and thus, 32 articles were included. The flow chart related to the identification and selection of the studies is shown in Fig. 1.

Tables 1 ^-^ 4 show the distribution of the studies according to the adopted characterization parameters. Of the 32 included articles,^11^^-^ 42 16 were developed in São Paulo,^27^^-^ 42 of which 10 in the capital.^33^^-^ 42 Only one study was carried out in the Midwest^17^ and North^19^ macro-regions each, whereas three studies were carried out in the South^12^^-^ 14 and the Southeast regions.^15^^-^ 17 Seven studies were systematized in Northeast.^20^^-^ 26 It is also noteworthy a study that included the overall nutritional assessment of the five geographical macro-regions of the country.^11^

**Table 1:** Characteristics of the observational articles on anthropometric assessment of Brazilian children attending daycare centers (national studies and studies from the South, Southeast, Midwest and North regions).

Author, year	Location	Assessment method	Reference standard	Prevalence (%)
Silva et al., 2000^11^	Brazil	NR	NCHS, 1977	W/A<-2Z: 5.4
				H/A<-2Z: 12.6
				W/H<-2Z: 1.3
Rodrigues et al., 2011^12^	Cascavel (PR)	Lohman et al., 1988	WHO, 2006	W/A<-2Z: 2.3
				W/H<-2Z: 0.4
				W/H>+2Z: 9.8
Dallabona et al., 2010^13^	Balneário Camboriú (SC)	NR	WHO, 2006	BMI/A<-2Z: 2.6
				BMI/A>+1Z: 29.6
				BMI/A>+2Z: 9.5
Corso et al., 2004^14^	Florianópolis (SC)	NR	NCHS, 1977	H/A<-2Z: 8.7
				W/H<-2Z: 1.1
				W/H>+2Z: 8.6
Rocha et al., 2008^15^	Belo Horizonte (MG)	WHO, 1995	CDC/NCHS, 2000	W/A<-2Z: 5.5
				H/A<-2Z: 4.2
				W/H<-2Z: 5.0
Camilo et al., 2008^16^	Guaxupé (MG)	Jelliffe, 1968	NCHS, 1977	H/A<-2Z: 3.3
Castro et al., 2005^17^	Viçosa (MG)	Jelliffe, 1968	NCHS, 1977	W/A<-2Z: 0.0
				H/A<-2Z: 3.5
				W/H<-2Z: 0.0
				W/H>+2Z: 4.6
Tuma et al., 2005^18^	Brasília (DF)	Jelliffe, 1968	NCHS, 1977	W/A<-2Z: 2.2
				H/A<-2Z: 4.8
				W/H<-2Z: 0.4
				W/H>+2Z: 6.1
Santos, 1999^19^	Capitão Poço (PA)	FAO/WHO, 1985	NCHS, 1977	W/A<-2Z: 53.0
				H/A<-2Z: 55.0

W/A, weight for age; H/A, height for age; W/H, weight for height; BMI/A, Body Mass Index for age; NR, not reported (unreferenced anthropometric method).

**Table 2:** Characteristics of observational articles on anthropometric assessment of Brazilian Children attending daycare centers (studies in the Northeast region).

Author, year	Location	Assessment method	Reference standard	Prevalence (%)
Pedraza et al., 2013^20^	Paraíba	WHO, 1995	WHO, 2006	H/A<-2Z: 7.4
				W/H<-2Z: 1.1
				W/H>+2Z: 6.2
Sousa et al., 2012^21^	João Pessoa (PB)	WHO, 1995	WHO, 2006	H/A<-2Z: 7.6
				W/H<-2Z: 1.6
				W/H>+2Z: 6.4
Pedraza et al., 2011^22^	Paraíba	WHO, 1995	WHO, 2006	H/A<-2Z: 7.7
Sousa et al., 2011^23^	Paraíba	WHO, 1995	WHO, 2006	H/A<-2Z: 5.8
				W/H<-2Z: 0.4
				W/H>+2Z: 3.8
Azevedo et al., 2010^24^	Recife (PE)	NR	WHO, 2006	W/A<-2Z: 2.5
				H/A<-2Z: 8.6
				W/H<-2Z: 1.5
Barreto et al., 2007^25^	Natal (RN)	NR	CDC/NCHS, 2000	BMI/A≥p85: 19.7
				BMI/A≥p95: 7.1
Cavalcanti et al., 2003^26^	12 municipalities in Paraíba (PB)	NR	NCHS, 1977	W/A<-2Z: 6.9

W/A, weight for age; H/A, Height for age; W/H, weight for height; BMI/A, Body Mass Index for age; NR, not reported (anthropometric method unreferenced).

**Table 3:** Characteristics of observational articles on anthropometric assessment of Brazilian children attending daycare centers (studies in other cities of São Paulo rather than the capital).

Author, year	Location	Assessment method	Reference standard	Prevalence (%)
Nascimento et al., 2012^27^	Taubaté	Lohman et al., 1988	WHO, 2006	H/A<-2Z: 2.9
				BMI/A<-2Z: 0.9
				BMI/A>+1Z: 28.9
				BMI/A>+2Z: 8.9
Almeida et al., 2007^28^	Jardinópolis	NR	CDC/NCHS, 2000	W/A<-2Z: 1.6
				H/A<-2Z: 0.5
				W/H<-2Z: 4.3
				W/H>+2Z: 2.2
Silva, 2004^29^	Piracicaba	NR	NCHS, 1977	H/A<-2Z: 7.0
Silva et al., 2000^30^	Embu	NR	NCHS, 1977	Malnourished (Gómez criteria for children <24 months and Waterlow criteria for children ≥24 months): 13.3
Silva e Sturion, 1998^31^	Piracicaba	NR	NCHS, 1977	H/A<-2Z: 5.1
				W/H<-2Z: 1.3
Antonio et al., 1996^32^	Paulínia	Jelliffe, 1966; Marshall, 1977; Cameron, 1978	NCHS, 1977	Malnourished (Gómez criteria): 21.0

W/A, weight for age; H/A, Height for age; W/H, weight for height; BMI/A, Body Mass Index for age; NR, not reported (unreferenced anthropometric method).

**Table 4:** Characteristics of observational articles on anthropometric assessment of Brazilian children attending daycare centers (studies in the capital city of the state of São Paulo).

Author, year	Assessment method	Reference standard	Prevalence (%)
Toloni et al., 2009^33^	NR	NCHS, 1977	W/A<-2Z: 4.4
Fujimori et al., 2007^34^	Ministry of Health, 2001	NCHS, 1977	W/A<-2Z: 0.7
			H/A<-2Z: 2.1
			W/H<-2Z: 2.7
			W/H>+2Z: 0.7
Bueno e Fisberg, 2006^35^	Habicht et al., 1974	WHO, 2006; CDC/NCHS, 2000; IOTF, 2000	W/H>+2Z: 6.2
			BMI/A≥p85: 22.5
			BMI/A≥p95: 9.3
Zöllner e Fisberg, 2006^36^	Lohman et al., 1988; Habicht et al., 1974	NCHS, 1977	W/A<-2Z: 3.1
			H/A<-2Z: 5.2
			W/H<-2Z: 0.9
			W/H>+2Z: 5.0
Fisberg et al., 2004^37^	Habicht, 1974; Lohman et al., 1998	NCHS, 1977	H/A<-2Z: 7.0
			W/H<-2Z: 0.9
Bueno et al., 2003^38^	Habicht et al., 1974	NCHS, 1977	W/A<-2Z: 2.9-1.7
			H/A<-2Z: 7.1-3.1
			W/H<-2Z: 0.2-0.5
			W/H>+2Z: 5.7-6.9
Prado et al., 2002^39^	NR	NCHS, 1977	W/A<-2Z: 1.5
Taddei et al., 2000^40^	NR	NCHS, 1977	W/A<-1Z: 29.8-15.2
			H/A<-1Z: 50.0-44.8
			W/H<-1Z: 10.1-3.4
Souza e Taddei, 1998^41^	NR	NCHS, 1977	W/A<-2Z: 2.8-1.4
			H/A<-2Z: 12.4-6.9
			W/H<-2Z: 1.4-0.0
			Malnourished (Gómez criteria): 31.0-17.2
Siviero et al., 1997^42^	NR	Marcones et al., 1982, Marques et al., 1982 and NCHS, 1977	W/A<-2Z: 2.8-1.2
			H/A<-2Z: 4.4-3.3
			W/H<-2Z: 0.4-0.3

W/A, weight for age; H/A, Height for age; W/H, weight for height; BMI/A, Body Mass Index for age; NR, not reported (unreferenced anthropometric method).

The anthropometric methods proposed by Jelliffe (1968), Habicht (1974), Lohman (1988) and the World Health Organization (WHO) (1995) were the most often used ones. Fujimori et al.^34^ based their study on the specific recommendations of the Brazilian Ministry of Health of 2001. The anthropometric assessment method was not reported in 15 of the reviewed studies.^11^^,^ 13 ^,^ 14 ^,^ 19 ^,^ 24 ^-^ 26 ^,^ 28 ^,^ 30 ^,^ 31 ^,^ 33 ^,^ 39 ^-^ 42

The reference standard of the National Centers for Health Statistics - NCHS (1977), recommended by the WHO, was used in 20 studies.^11^^,^ 14 ^,^ 16 ^-^ 19 ^,^ 26 ^,^ 29 ^-^ 34 ^,^ 36 ^-^ 42 The standard of the Centers for Disease Control-CDC/NCHS (2000) was used in four studies.^15^^,^ 25 ^,^ 28 ^,^ 35 One study^35^ compared the results of three nutritional status classification criteria: NCHS (1977), CDC/NCHS (2000), International Obesity Task Force-IOTF (2000), WHO (2006). The WHO growth curves, of which first communications occurred in 2004 and were distributed in 2006, were used in all articles published from the year 2010 on.

A total of 11 studies^15^^,^ 17 ^,^ 18 ^,^ 24 ^,^ 28 ^,^ 30 ^,^ 34 ^,^ 36 ^,^ 38 ^,^ 41 ^,^ 42 considered the anthropometric indices height for age (H/A), weight for height (W/H) and weight for age (W/A) to assess children's nutritional status. The H/A was also the diagnostic object in 11 other studies,^14^^,^ 16 ^,^ 20 ^-^ 23 ^,^ 27 ^,^ 29 ^,^ 37 the W/H in eight studies^12^^,^ 14 ^,^ 16 ^,^ 20 ^,^ 21 ^,^ 23 ^,^ 31 ^,^ 35 ^,^ 37and W/A in six studies.^12^^,^ 19 ^,^ 26 ^,^ 32 ^,^ 33 ^,^ 39

The prevalence of malnutrition and overweight expressed by the standard deviation of the indexes H/A (<−2 *z* score, chronic malnutrition indicator), W/H (<−2 *z* score, acute malnutrition indicator; >+2 *z* score, indicator of overweight/obesity) and W/A (<−2 *z* score, overall malnutrition indicator) varied widely. For H/A index, the prevalence of children with stunting ranged from 0.5%^28^ to 55%,^19^ according to the studies in the municipalities of Jardinópolis (SP) and Capitão Poço (PA), respectively. As for the W/H index, the prevalence of children with acute malnutrition ranged between 0%^17^ and 5%,^15^ according to the studies carried out in Viçosa (MG) and Belo Horizonte (MG), respectively. The prevalence of overweight children ranged from 0.7%, a reference value for children from São Paulo (SP)^34^ to 9.8%, the reference value for children from Cascavel (PR).^12^ Regarding the W/A index, the prevalence of children with values <−2 *z*-score ranged between 0%^17^ and 53%.^19^

The classification of Gómez (1955), Waterlow (1977) and the Body Mass Index for age (BMI/A) was also used to indicate malnutrition. Silva et al.,^30^ Souza and Taddei^41^ and Antonio et al.^32^ showed malnutrition prevalence between 13.3%^30^ and 31%^41^ when using the Gómez and/or Waterlow classifications. The studies using BMI/A^13^^,^ 25 ^,^ 27 ^,^ 35 reported overweight prevalence between 19.7%^25^ and 29.6%.^13^

In more recent studies, published from 2010 on and that used the WHO growth curves as references, acute malnutrition ranged between 0.4%^12^^,^ 23 and 1.8%,^24^ and stunting ranged from 2.9%^27^ to 8.6%.^24^ Overweight, for this set of articles, showed prevalence between 22.5%^35^ and 29.6%,^13^ according to the BMI/A, and between 3.8%^23^ and 9.8%,^12^ according to the W/H.

## Discussion

Regarding the anthropometric assessment method, in spite of the numerous references used in the systematized articles, it is possible to assume standardization. The anthropometric methods appeared with the publications by Jellife in the 60s, systematizing a same technique.^43^ Since then, anthropometrics developed constantly, making it possible to advance in the interpretation and the search for mathematical formulations with improved accuracy in body compartment estimation and their predictive power. Thus, anthropometrics has been shown to be the most often used isolated method for nutritional diagnosis at the population level, especially in childhood and adolescence, due to being easy to perform, in addition to its low cost and safety.^2^^,^ 43

Anthropometric values represent, at the individual or population level, the degree of adjustment between the genetic growth potential and the beneficial and adverse environmental factors. There is evidence that the height and weight of healthy children from different ethnic backgrounds, submitted to appropriate living conditions, are similar up to the age of five years. Thus, it is possible to use a single international standard to assess growth and nutritional status in different regions. Therefore, the WHO adopted, since 1978, the NCHS data as the international reference standard, later reformulated as the CDC/NCHS reference standard (2000).^43^ The use of this reference in all studies of this review reflects its global acceptance.

The need to construct a new growth curve of children and adolescents appeared in 1995. Among other views, it was deemed important to consider aspects such as breastfeeding (children in the NCHS curves were formula-fed), inclusion of other anthropometric indicators and use of data from other countries (children in the NCHS curves were only from the US).^44^ These curves were publicly presented in 2006 and their use is recommended by the Ministry of Health of Brazil.^2^ However, it can be observed in the studies included in this present review that the nutritional assessment of children attending daycare centers, using the latter reference, was consolidated only in the articles published from 2010 to the present date. This fact can be explained by the fact that it was impossible for the authors to use this reference standard considering the proximity between the year of publication of the new growth curves (2006), or its recommendation by the Ministry of Health (2008), and the date of submission/acceptance/publication of the articles in this review published between 2006 and 2013.

The anthropometric classifications by Gómez,^45^ Waterlow^46^ and WHO^47^ have been the most widely used over time. Although they are no longer recommended, the classifications by Gómez and Waterlow were used in three review studies.^30^^,^ 32 ^,^ 41. The WHO^47^ criteria are still currently used. These criteria establish the comparison of anthropometric measurements with the reference standard through the use of scales, of which the most common is the percentile and standard deviation (or *z* score: number of standard deviations that the obtained data is deviated from its reference median).^43^ The *z*-score calculation of the H/A, W/H and W/A indexes in all studies of this review that used the WHO criteria, suggests the preference and predominance of these parameters as malnutrition cutoffs, as recommended by the WHO.^47^

In order to perform the analysis of several studies, their comparability should be assumed. This comparability depends, among other factors, on the methods used in the anthropometric assessment, on the study location/population and time. From a methodological point of view, the comparability possibility was previously discussed regarding the use of the same indicators, reference standards, cutoffs and techniques that allow reducing possible variations in the quantification of malnutrition cases. In this context, it is important to note that the Gómez and Waterlow classifications have essentially different and non-comparable classification criteria from a methodological point of view, with the NCHS and CDC/NCHS growth curves.

However, in addition to the restrictive nature of the studies (although comparable, they are pointwise investigations) and the concentration of studies carried out in São Paulo, with scarcity in other areas of Brazil, there is an important factor of dispersion of results associated with time (18 years for 33 studies), which brings restrictions to study comparability. The differences between the nutritional deficit prevalence rates have changed significantly between studies, which may be due to the daycare center location, the children's socioeconomic status, but also the time of data collection. For instance, the H/A ratio ranged from 0.5% to 55%, but one study was carried out in 1999 in Pará and the other one in 2007 in São Paulo. It is known that malnutrition is decreasing with time, in a dynamic process of nutritional transition, which has changed the nutritional scenario in Brazil. Therefore, conclusions about the prevalence of malnutrition using study data collected at different times can show a bias, as the older data no longer represent the children's nutritional status. The results are, in fact, diachronic. The analysis of variations in nutritional status indicators must face similar difficulties. Finally, the situation that encompasses a long time and diverse geographical areas produces a dispersion that needs to be considered to outline the anthropometric profile of Brazilian children attending daycare centers; however, its adjustment is a difficult one.

If the previous limitation can be clearly observed in all the articles reviewed herein, it is also observed among the studies^12^^,^ 13 ^,^ 20 ^-^ 24 ^,^ 27 ^,^ 35 that considered as a reference standard the infant growth curves by WHO 2006,^48^ that most were published from 2010 onward, with plausible synchronous results of systematization. In these studies, the prevalence of malnutrition indicated by the W/H ratio is low, between 0.4%^12^^,^ 23 and 1.8%,^24^ values that indicate virtually no risk of malnutrition because they represent similar frequencies to those found in the reference distribution.^20^ The assessed children's overweight can also be verified considering all of these articles, according to both the BMI/A, which reports prevalence between 22.5%^35^ and 29.6%,^13^ as well as the W/H ratio, with numbers ranging from 3.8%^23^ to 9.8%.^12^ Considering the stunting prevalence, we have a weighted mean through the respective sample sizes of 6.3% (range: 8.6-2.9), which corresponds to 125 children with short stature of a total of 1969 children, indicating significant rates, either considering the distribution of the reference population or the WHO parameters to classify the severity of the problem.^20^

These analyses suggest a high prevalence of overweight, no acute malnutrition, and a still significant prevalence of stunting, indicating the occurrence of a nutritional transition process in the population of Brazilian children attending daycare centers. Nationwide Brazilian surveys,^49^^,^ 50 which have used the new growth curves for the analysis of the nutritional status of children younger than five years, have shown similar findings. The Household Budget Survey (2008-2009)^50^ results, moreover, showed that the prevalence of stunting in children younger than five years old varies according to the income class, from 8.2% when the family monthly *per capita* income is up to ¼ of a minimum wage, to 3.1%, when the household income is more than five minimum wages. In this context, there is evidence of a greater potential vulnerability of children attending daycare centers, since this is reality is mainly associated to a lack of resources or because the children's mothers work outside the home.^51^

It is noteworthy that the results analyzed here represent the reality of children attending public day care centers, as, in their studies, the researchers predominantly analyzed daycare centers of public administration. Only two studies included childcare institutions administered by the private sector,^13^^,^ 29 which were published in 2004^29^ and 2010.^13^ Therefore, the systematized results refer to children from a vulnerable socioeconomic class, whose families need the services provided by public daycare centers, either municipal or state-run institutions. Also, this review included articles identified in only three bibliographic databases, which can limit the analysis spectrum (possibility of limited number of cases/institutions not knowing about the representativeness).

Although the results of this article suggest that the nutritional transition process observed in the Brazilian population is also present in the population of children attending daycare centers, it emphasizes the need to develop a multicenter survey on health and nutrition, combined with a higher number of pointwise investigations, but comparable, aiming to more accurately assess the current behavior of malnutrition prevalence in children attending daycare centers. This would allow a clearer comparison of the nutritional status indicators in children attending daycare centers with national data and from other vulnerable groups, as well as the planning of interventions aimed at controlling overweight and stunting.

## References

[1] Machado PG, Mezzomo CL (2011). A relação da postura corporal, da respiração oral e do estado nutricional em crianças - uma revisão de literatura. Rev CEFAC.

[2] Araújo AC, Campos JA (2008). Subsídios para a avaliação do estado nutricional de crianças e adolescentes por meio de indicadores antropométricos. Alim Nutr.

[3] Pereira AS, Lanzillotti HS, Soares EA (2010). Frequência à creche e estado nutricional de pré-escolares: uma revisão sistemática. Rev Paul Pediatr.

[4] De Onis M, Blössner M, Borghi E (2012). Prevalence and trends of stunting among pre-school children, 1990-2020. Public Health Nutr.

[5] Lima AL, Silva AC, Konno SC, Conde WL, Benicio MH, Monteiro CA (2010). Causas do declínio acelerado da desnutrição infantil no Nordeste do Brasil (1986-1996-2006). Rev Saude Publica.

[6] Monteiro CA, Benicio MH, Konno SC, Silva AC, Lima AL, Conde WL (2009). Causes for the decline in child under-nutrition in Brazil, 1996-2007. Rev Saude Publica.

[7] Goulart RM, Banduk ML, Taddei JA (2010). Uma revisão das ações de nutrição e do papel do nutricionista em creches. Rev Nutr.

[8] Bógus CM, Nogueira-Martins MC, Moraes DE, Taddei JA (2007). Cuidados oferecidos pelas creches: percepções de mães e educadoras. Rev Nutr.

[9] Figueroa Pedraza D, Queiroz D, Sales MC (2014). Doenças infecciosas em crianças pré-escolares brasileiras assistidas em creches. Cienc Saude Colet.

[10] Segall-Corrêa AM, Gonçalves NN, Chalita LV, Russo-Leite GP, Padovani CR, Gonçalves A (2002). Determinantes da evolução do peso e altura em crianças de 3 meses a 6 anos assistidas em creche: análise por modelo linear não hierarquizado em ensaio quase-experimental. Rev Panam Salud Publica.

[11] Silva MV, Ometto AM, Furtuoso MC, Pipitone MA, Sturion GL (2000). Acesso à creche e estado nutricional das crianças brasileiras: diferenças regionais, por faixa etária e classe de renda. Rev Nutr.

[12] Rodrigues VC, Mendes BD, Gozzi Aline Sandrini F, Santana RG, Matioli G (2011). Deficiência de ferro, prevalência de anemia e fatores associados em crianças de creches públicas do oeste do Paraná, Brasil. Rev Nutr.

[13] Dallabona A, Cabral SC, Höfelman DA (2010). Variáveis infantis e maternas associadas à presença de sobrepeso em crianças de creches. Rev Paul Pediatr.

[14] Corso AC, Viteritte PL, Peres MA (2004). Prevalência de sobrepeso e sua associação com a área de residência em crianças menores de 6 anos de idade matriculadas em creches públicas de Florianópolis, Santa Catarina, Brasil. Rev Bras Epidemiol.

[15] Camillo CC, Amancio OM, Vitalle MS, Braga JA, Juliano Y (2008). Anemia ferropriva e estado nutricional de crianças de creches de Guaxupé. Rev Assoc Med Bras.

[16] Rocha DS, Lamounier JA, Capanema FD, Franceschini SC, Norton RC, Costa AB (2008). Estado nutricional e prevalência de anemia em crianças que frequentam creches em Belo Horizonte, Minas Gerais. Rev Paul Pediatr.

[17] Castro TG, Novaes JF, Silva MR, Costa NM, Franceschini SC, Tinoco AL (2005). Caracterização do consumo alimentar, ambiente socioeconômico e estado nutricional de pré-escolares de creches municipais. Rev Nutr.

[18] Tuma RC, Costa TH, Schimitz BA (2005). Avaliação antropométrica e dietética de pré-escolares em três creches de Brasília, Distrito Federal. Rev Bras Saude Mater Infant.

[19] Santos MF (1999). Perfil antropométrico de crianças de 01 a 07 anos de idade do município de Capitão Poço-Pará-Brasil. Rev Para Med.

[20] Figueroa Pedraza D, Rocha AC, Sousa CP (2013). Crescimento e deficiências de micronutrientes: perfil das crianças assistidas no núcleo de creches do governo da Paraíba, Brasil. Cienc Saude Colet.

[21] Souza MM, Figueroa Pedraza D, Menezes TN (2012). Estado nutricional de crianças assistidas em creches e situação de (in)segurança alimentar de suas famílias. Cienc Saude Colet.

[22] Figueroa Pedraza D, Rocha AC, Queiroz EO, Sousa CP (2011). Estado nutricional relativo ao zinco de crianças que frequentam creches do estado da Paraíba. Rev Nutr.

[23] Sousa CP, Sousa MP, Rocha AC, Figueroa Pedraza D (2011). Perfil epidemiológico do estado nutricional de crianças assistidas em creches no Estado da Paraíba. Nutrire: Rev Soc Bras Alim Nutr.

[24] Sales de Azevedo MM, Cabral PC, Diniz AS, Fisberg M, Fisberg RM, Arruda IK (2010). Deficiência de vitamina A em pré-escolares da cidade do Recife, nordeste do Brasil. Arch Latinoam Nutr.

[25] Barreto AC, Brasil LM, Maranhão HS (2007). Sobrepeso: uma nova realidade no estado nutricional de pré-escolares de Natal, RN. Rev Assoc Med Bras.

[26] Cavalcanti CL, Gonçalves VB, Valença AM, Cavalcanti AL, Vieira RK (2003). Estado nutricional de pré-escolares e valor nutricional da merenda escolar oferecida em creches públicas da Paraíba-PB. Pesq Bras Odontoped Clin Integr.

[27] Nascimento VG, Silva JP, Bertoli CJ, Abreu LC, Valenti VE, Leone C (2012). Prevalência de sobrepeso em crianças pré-escolares em creches públicas: um estudo transversal. São Paulo Med J.

[28] Almeida CA, Ramos AP, João CA, João CR, Ricco RG, Dutra-de-Oliveira JE (2007). Jardinópolis sem anemia primeira fase: avaliação antropométrica e do estado nutricional de ferro. Rev Paul Pediatr.

[29] Silva MV (2004). A frequência à creche influencia o estado nutricional infantil?. Nutrire: Rev Soc Bras Alim.

[30] Silva EM, Miranda CT, Puccini RF, Nóbrega FJ (2000). Day care centres as an institution for health promotion among needy children: an analytical study in São Paulo, Brazil. Public Health.

[31] Silva MV, Sturion GL (1998). Frequência à creche e outros condicionantes do estado nutricional infantil. Rev Nutr.

[32] Antonio MA, Morcillo AM, Piedrabuena AE, Carniel EF (1996). Avaliação nutricional das crianças matriculadas nas quatorze creches municipais de Paulínia, SP. Rev Paul Pediatr.

[33] Toloni MH, Konstantyner T, Taddei JA (2009). Fatores de risco para perda ponderal de crianças frequentadoras de berçários em creches do município de São Paulo. Rev Paul Pediatr.

[34] Fujimori E, Palombo CN, Schoeps FA, Duarte LS (2007). Perfil nutricional e de morbidade de crianças atendidas em uma creche pública. REME.

[35] Bueno MB, Fisberg RM (2006). Comparação de três critérios de classificação de sobrepeso e obesidade entre pré-escolares. Rev Bras Matern Infant.

[36] Zöllner CC, Fisberg RM (2006). Estado nutricional e sua relação com fatores biológicos, sociais e demográficos de crianças assistidas por creches da Prefeitura do Município de São Paulo. Rev Bras Saude Matern Infant.

[37] Fisberg RM, Marchioni DM, Cardoso MR (2004). Estado nutricional e fatores associados ao déficit de crescimento de crianças frequentadoras de creches públicas do Município de São Paulo, Brasil. Cad Saude Publica.

[38] Bueno MB, Marchioni DM, Fisberg RM (2003). Evolução nutricional de crianças atendidas em creches públicas no Município de São Paulo, Brasil. Rev Panam Salud Publica.

[39] Prado SR, Sigulem DM, Juliano Y, Cury MC (2002). Razão de risco de morbidade e estado nutricional em crianças de creche. Rev Paul Pediatr.

[40] Taddei JA, Cannon MJ, Warner L, Souza P, Vitalle S, Palma D (2000). Nutritional gains of underprivileged children attending a day care center in S. Paulo City, Brazil: a nine month follow-up study. Rev Bras Epidemiol.

[41] Souza PC, Taddei JA (1998). Efeito da frequência à creche nas condições de saúde e nutrição de pré-escolares residentes em favelas da periferia de São Paulo, 1996. Rev Paul Pediatr.

[42] Siviero AA, Anti SM, Bandeira CM, Russeff MM, Fisberg M (1997). Intervenção e orientação nutricional no acompanhamento de crianças desnutridas em creches de São Paulo. Rev Paul Pediatr.

[43] Sigulem DM, Devincenzi MU, Lessa AC (2000). Diagnóstico do estado nutricional da criança e do adolescente. J Pediatr (Rio J).

[44] Zeferino AM, Barros AA, Bettiol H, Barbieri MA (2003). Acompanhamento do crescimento. J Pediatr (Rio J).

[45] Gomez F, Galvan RR, Cravioto J, Frenk S (1955). Malnutrition in infancy and childhood, with special reference to Kwashiorkor. Adv Pediatr.

[46] Waterlow JC, Buzina R, Keller W, Lane JM, Nichaman MZ, Tanner JM (1977). The presentation and use of height and weight data for comparing the nutritional status of groups of children under the age 10 years. Bull WHO.

[47] World Health Organization (1995). The use and interpretation of anthropometry: report of a WHO Expert Committee.

[48] World Health Organization (2006). Child growth standard.

[49] Brasil - Ministério da Saúde (2008). Pesquisa nacional sobre demografia e saúde da criança e da mulher.

[50] Brasil - Instituto Brasileiro de Geografia e Estatística (2010). Pesquisa de Orçamentos Familiares 2008-2009: Antropometria e estado nutricional de crianças, adolescentes e adultos no Brasil.

[51] Oliveira MN, Taddei JA, Taddei JA, Longo-Silva G, Toloni MH (2011). Terceirização dos cuidados com crianças na sociedade contemporânea.

